# Air-Coupled Low Frequency Ultrasonic Transducers and Arrays with PMN-32%PT Piezoelectric Crystals

**DOI:** 10.3390/s17010095

**Published:** 2017-01-06

**Authors:** Rymantas J. Kazys, Reimondas Sliteris, Justina Sestoke

**Affiliations:** Ultrasound Institute, Kaunas University of Technology, Kaunas LT-51423, Lithuania; reimondas.sliteris@ktu.lt (R.S.); justina.sestoke@ktu.lt (J.S.)

**Keywords:** ultrasonic transducer, air coupled, PMN-32%PT crystals, ultrasonic array

## Abstract

Air-coupled ultrasonic techniques are being increasingly used for material characterization, non-destructive evaluation of composite materials using guided waves as well as for distance measurements. Application of those techniques is mainly limited by the big losses of ultrasonic signals due to attenuation and mismatch of the acoustic impedances of ultrasonic transducers and air. One of the ways to solve this problem is by application of novel more efficient piezoelectric materials like lead magnesium niobate-lead titanate (PMN-PT) type crystals. The objective of this research was the development and investigation of low frequency (<50 kHz) wide band air-coupled ultrasonic transducers and arrays with an improved performance using PMN-32%PT crystals. Results of finite element modelling and experimental investigations of the developed transducers and arrays are presented. For improvement of the performance strip-like matching elements made of low acoustic impedance, materials such as polystyrene foams were applied. It allowed to achieve transduction losses for one single element transducer −11.4 dB, what is better than of commercially available air-coupled ultrasonic transducers. Theoretical and experimental investigations of the acoustic fields radiated by the eight element ultrasonic array demonstrated not only a good performance of the array in a pulse mode, but also very good possibilities to electronically focus and steer the ultrasonic beam in space.

## 1. Introduction

Air-coupled ultrasonic techniques are already used for range finding and surface profile measurements [1,2], material characterization, non-destructive evaluation (NDE) [3,4,5,6,7,8,9,10,11] and even for secure wireless transmission of data [12].

The frequency range of the air-coupled transducers used depends on a particular application and properties of the materials under investigation and environment. For distance measurements usually rather low frequencies (<100 kHz) are exploited, except for surface profile measurements in which the frequency range may reach 500–1000 kHz.

In the NDE field, air-coupled techniques were applied for inspection not only of low acoustic impedance materials such as composites [3,4], but also of metals [5]. In order to get a better spatial resolution and accuracy high frequency ultrasonic waves up to 1200 kHz are used [3,6]. Air-coupled techniques are already widely exploited for contactless excitation and reception of guided waves in composite structures [7,8,9,10,11]. For this purpose, usually the lowest A_0_ Lamb wave mode is used, which is easily excited when the ultrasound velocity in air is slower than the phase velocity of the A_0_ mode. In this case, the Lamb wave is a leaky wave, which is excited and picked up by air-coupled ultrasonic transducers, deflected with respect to the surface of the structure according to the Snell’s law.

The velocity of the A_0_ mode is frequency dependent and at some frequency becomes slower than the ultrasound velocity in air. In this case, the A_0_ Lamb wave propagates only inside the structure under investigation and no leaky wave is radiated into surrounding air. Such a wave is called an evanescent Lamb wave. Such waves may be very attractive for NDE of composite structures of big dimensions, because they may propagate without significant losses. However, the main problem is how to excite and pick up such waves using the air-coupled approach because according to Snell’s law it should be impossible. That is because according to Snell’s law, when the ultrasound velocity in the structure is lower than in air the required incident angle of the air-coupled wave does not exist, e.g., it is not physically feasible. We have shown that this kind of excitation and reception may be achieved using low frequency (<50 kHz) air-coupled ultrasonic arrays [13]. Therefore, development of relatively low frequency efficient air-coupled ultrasonic transducers and arrays is important for NDE of composite materials using guided waves, as well as for distance measurement applications.

The main problem met in air-coupled ultrasonics is the signal losses caused by attenuation and significant mismatch of the acoustic impedances of ultrasonic transducers and air. One solution to this problem is application of 1–3 connectivity type composite piezoelectric elements, the specific acoustic impedance of which is lower than that of monolithic piezoelectric ceramics [14]. Acoustic matching layers made of materials with low acoustic impedance [15,16,17] or using a thin membrane in front of a piezoelectric element [18] may improve performance of piezoelectric transducers.

However, application of monolithic matching layers in ultrasonic arrays is not suitable, because it will create cross-talk between elements of the array. Further enhancement of the performance of ultrasonic air transducers is limited by the piezoelectric coupling coefficients of the commercially available piezoelectric materials.

The advent of novel piezoelectric materials with very strong piezoelectric effects such as lead magnesium niobate-lead titanate [PbMg_1/3_Nb_2/3_O_3_-PbTiO (PMN-PT)] crystals enables one to improve the performance of ultrasonic transducers. The electromechanical coupling factors of PMN-32%PT crystals are very high. The electromechanical coupling factor for the thickness extension mode is *k*_33_ > 0.90, for the transverse shear mode *k*_15_ > 0.95 and for the transverse extension *k*_32_ > (0.84–0.90) [19,20,21]. The properties of the PMN-32%PT crystals may be further improved using domain engineering [22]. These parameters are higher than the similar parameters of piezoelectric ceramic elements used as active elements in ultrasonic transducers. PMN-32%PT crystals are already used in ultrasonic single crystal transducers and phased arrays for medical applications [23,24], in ultrasonic transducers for non-destructive testing [25] and ultrasonic hydrophones [26]. Application of the PMN-32%PT crystals for air-coupled ultrasonic transducers was suggested in our previous works [27,28,29]. However, to our knowledge, PMN-32%PT piezoelectric elements have not been yet systematically investigated from the point of view of their application for air-coupled ultrasonic transducers and arrays. The objective of this research was therefore the development of low frequency (<50 kHz) wide band air-coupled ultrasonic transducers and arrays with improved performance using PMN-32%PT crystals.

## 2. Properties of PMN-32%PT Crystals

In order to generate and pick up relatively low-frequency (<100 kHz) ultrasonic waves in air a suitable vibration mode of PMN-32%PT crystals should be selected. For this purpose, a transverse extension mode may be used in which displacements are excited in a plane perpendicular to a poling direction. In this case, the resonance frequency depends on the lateral dimensions of the crystal and may be quite low. The electric field is applied across the plate collinearly with the poling direction.

**Figure 1:**
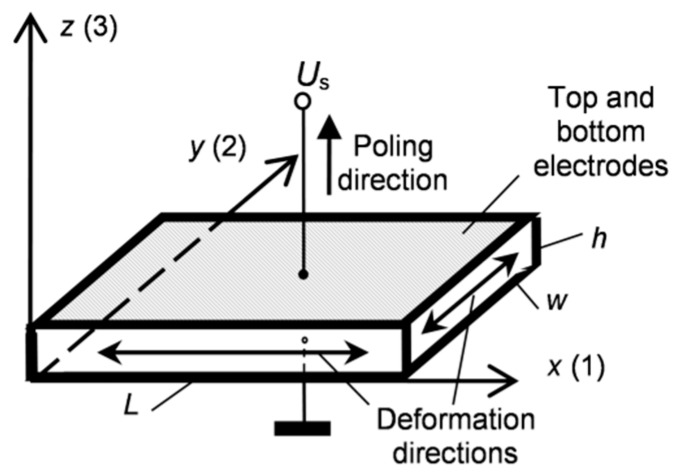
The plate vibrating in the transverse extension mode.

Such a mode can be excited in rectangular PMN-32%PT crystal plates with <011> cut and [001] poling direction (Figure 1). Another reason to select such a cut is that for the transverse extension mode the electromechanical coupling factors are high, for example, 0.77–0.90. This is about two times higher than for the <001> cut (*k*_3*j*_ = 0.43, *j* = 1, 2.) [19]. Square PMN-32%PT single crystals plates which are anisotropic, e.g., the piezoelectric properties in the directions of *x*(1) and *y*(2) axes are different, are commercially available (from HC Materials Corporation, Bolingbrook, IL, USA). We have performed measurements of electromechanical coupling coefficients, which were determined from the measured resonance *f*_r_ and antiresonance *f*_a_ frequencies of the appropriate vibration modes. The measurements of eight 15 × 15 × 1 mm^3^ crystal plates were carried out by the electric impedance meter (6500B, Wayne Kerr Electronics, West Sussex, UK). The main resonance frequency *f*_r2_ of the transverse extension mode in the direction *y*(2) is 37.5 kHz and in the direction *x*(1) is *f*_r1_ = 69.2 kHz. The measured electromechanical coupling coefficients were the following: *k*_31_ = 0.77 and *k*_32_ = 0.84–0.88, e.g., much higher than of piezo ceramic elements used in ultrasonic transducers.

**Figure 2:**
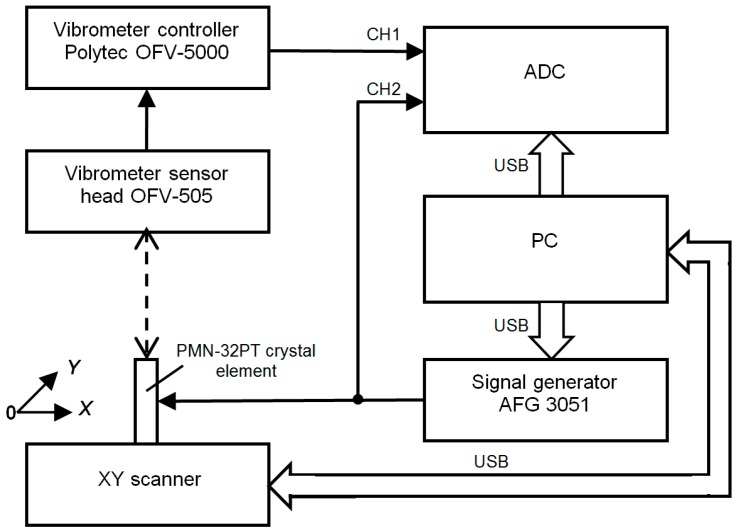
System for measurement of spatial distributions of normal displacements with the laser interferometer OFV-5000.

The biggest displacements take place in *x*0*y* plane of the crystal plate, e.g., in the *x* and *y* directions (Figure 1). In order to determine on which edge the biggest displacements are obtained, absolute measurements of the displacements at the main resonance frequency *f*_r2_ of the transverse extension mode were performed using a laser interferometer (OFV-5000, Polytec GmbH, Waldbronn, Germany, Figure 2).

**Figure 3:**
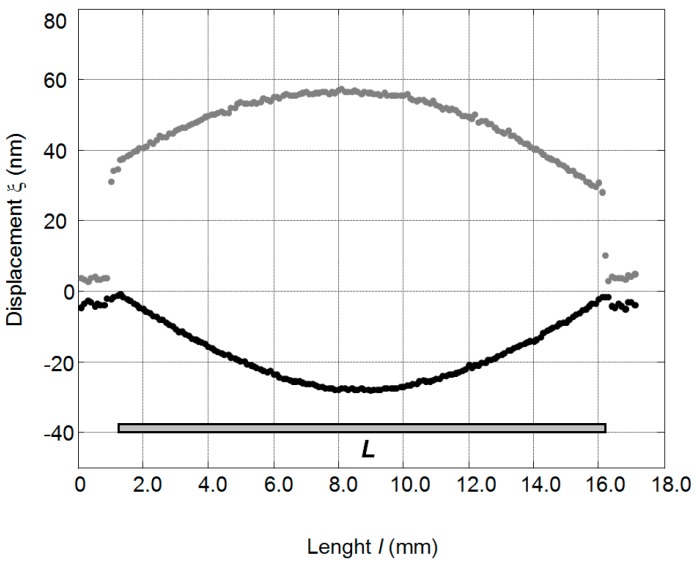
Displacements of the 15 × 15 × 1 mm^3^ PMN-32%PT crystal edges at the frequency *f*_0_ = 37.3 kHz, when the amplitude of the exciting signal is *U*_pp_ = 400 mV: *L*—length of the crystal edge; direction 2—grey; direction 1—black.

The measured spatial distributions of the active surfaces displacements of the PMN-PT 15 × 15 × 1 mm^3^ crystal at the resonance frequency *f*_0_ = 37.3 kHz are presented in Figure 3. From the results presented it follows that those distributions are non-uniform. It should be noted that displacements in the *x*(1) direction are of the opposite polarity to the displacements in the *y*(2) direction and are smaller.

From the measurements performed it follows that the maximal mechanical displacement is obtained at the main resonance frequency in the direction of *y*(2) axis [13,14,15]. Therefore, the edges of the crystal perpendicular to the *y*(2) direction were selected as the active surfaces used for radiation of ultrasonic waves.

## 3. Geometry of the Transducers and Arrays

**Figure 4:**
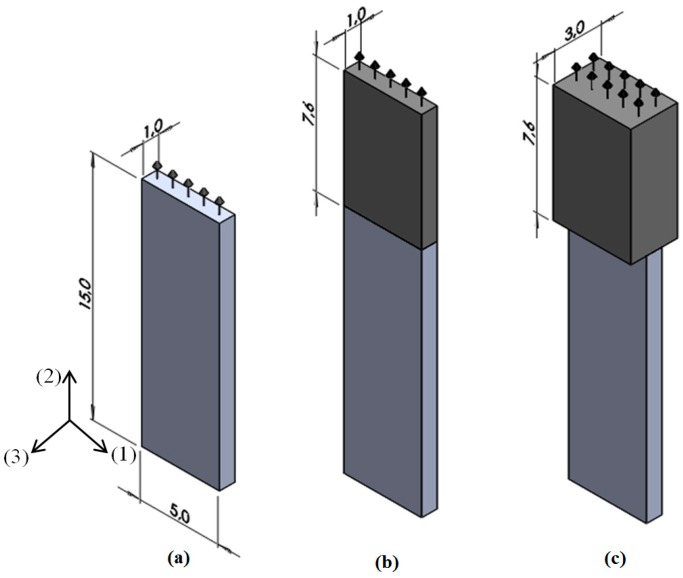
Air-coupled ultrasonic transducers (arrows indicate radiation of the active aperture): (**a**)—single PMN-32%PT element; (**b**)—the single element with the matching strip (darker colour, thickness *w* = 1 mm); (**c**)—single element with the matching strip (thickness *w* = 3 mm).

In order to get a more uniform distribution of the displacements on the active surface used for radiation of ultrasonic waves the square PMN-32%PT single crystal with dimensions 15 × 15 × 1 mm^3^ was cut into narrower rectangular strips with dimensions 15 × 5 × 1 mm^3^ and for radiation of ultrasonic waves the edge perpendicular to the *y*(2) direction was selected (Figure 4).

For improvement of the performance, special acoustic matching elements made of AIREX low impedance polystyrene foams were bonded to the active edge of the crystal. It should be noted, that conventional planar matching layers used in higher frequency transducers are not suitable in this case due to a selected geometry of the piezoelectric elements because the planar matching layer would create strong cross-talk between array elements. Therefore as matching elements not plates but strips made of polystyrene foam were proposed. The aperture used for radiation of ultrasonic waves in this case is rather small, therefore we investigated the performance of a few matching strips with different thicknesses *w*.

**Figure 5:**
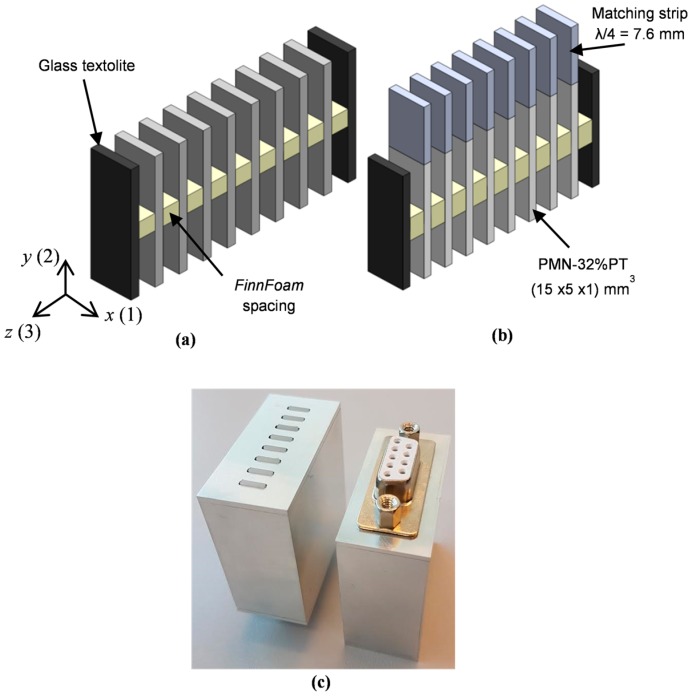
Air-coupled ultrasonic array: (**a**)—without matching strips; (**b**)—with the matching strips; (**c**)—the prototype of the air-coupled ultrasonic array.

On the other hand, the aperture may be increased by an ultrasonic array consisting of strip-like PMN-32%PT elements (Figure 5a). The ultrasonic array was assembled from individual piezoelectric strips separated by insulating elements. In this case, a bigger rectangular aperture of the transducers is obtained. The pitch between individual elements of the array was selected slightly less than λ_a_/2, where λ_a_ is the wavelength in air at the operation frequency. This gives the possibility to control electronically the radiated ultrasonic field in air. In order to improve performance of the array piezoelectric elements with matching strips (Figure 5b) are used.

The necessary distance between particular array elements is supported by spacing elements (Figure 5). The spacing elements are made of a low-density ρ = 38 kg/m^3^ Finnfoam material (Finnfoam OY, Salo, Finland) with dimensions 2 × 3 × 5 mm^3^. The velocity of ultrasonic longitudinal waves in Finnfoam materials was not known therefore we measured it by a through-transmission pulse method at the frequency 50 kHz in specially prepared samples. We have found that velocity is very low (*c* = 326 m/s) and correspondingly the acoustic impedance *Z* = 0.0124 MRayl is also low. Such properties of this material enable us to minimize acoustic cross-talk between the piezoelectric elements in the array.

The strip-like PMN-32%PT elements for the ultrasonic array were cut from the PMN-32%PT crystals with dimensions 15 × 15 × 1.0 mm^3^ (HC Materials Corporation, Bolingbrook, IL, USA) by a NanoAce-300e dicing machine (Loadpoint Ltd., Swindon, UK). The matching strips made of the AIREXT90.210 type polystyrene foam were bonded to the edges of PMN-PT crystal elements by a cyanoacrylate type glue (Fixpoint, Braunschweig, Germany). The assembly of the active elements into the array set was performed using the Finnfoam spacers and a polyvinyl acetate (PVA)-type glue. Glass textolite plates glued additionally from both sides of the array act as to fix the whole transducer assembly in the housing made of aluminium. The active PMN-32%PT elements are connected to a D-Sub 9P type connector.

## 4. Investigation of Air-Coupled PMN-32%PT Single Element Transducers

Vibrations and frequency responses of single PMN-32%PT element transducers were investigated both numerically and experimentally. Theoretical analysis was based on finite elements modelling (FEM) which was performed using the ANSYS Mechanical APDL software with SOLID5 elements with piezoelectric properties. The piezoelectric SOLID5 element has eight nodes with four nodal degrees of freedom: *x*, *y*, *z* directions and electric voltage *U*. The peak-to-peak amplitude of the electric excitation voltage was set *U*_p-p_ = 1 V.

**Figure 6:**
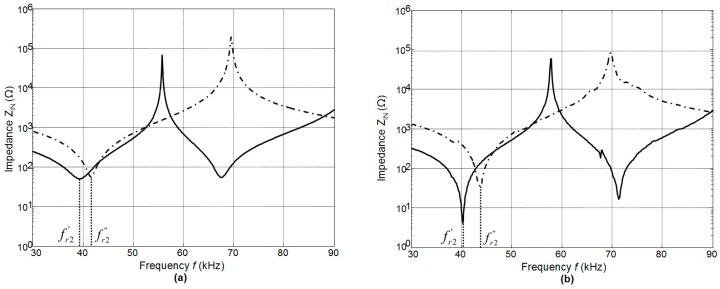
Electric input impedances of the single PMN-32%PT crystals: 15 × 15 × 1 mm^3^—solid line; 15 × 5 × 1 mm^3^—dashed line: (**a**)—calculated by the FEM; (**b**)—measured by the impedance meter.

The calculated electric input impedances of single PMN-32%PT crystals without matching strips with different dimensions 15 × 15 × 1 mm^3^ (solid line) and 15 × 5 × 1 mm^3^ (dashed line) are presented in Figure 6a. The resonance frequencies of the main longitudinal-extension mode of quadratic and strip-like elements are different—*f*^’^_r2_ = 39.3 kHz in the case of the square element and *f*^’’^_r2_ = 41.6 kHz of the strip-like element. The results of the measurements performed by an electric impedance meter (6500B, Wayne Kerr Electronics, West Sussex, UK) are shown in Figure 6b. The resonance frequencies are *f*^’^_r2_ = 40.3 kHz and *f*^’’^_r2_ = 43.7 kHz, e.g., rather close to the simulation results.

**Figure 7:**
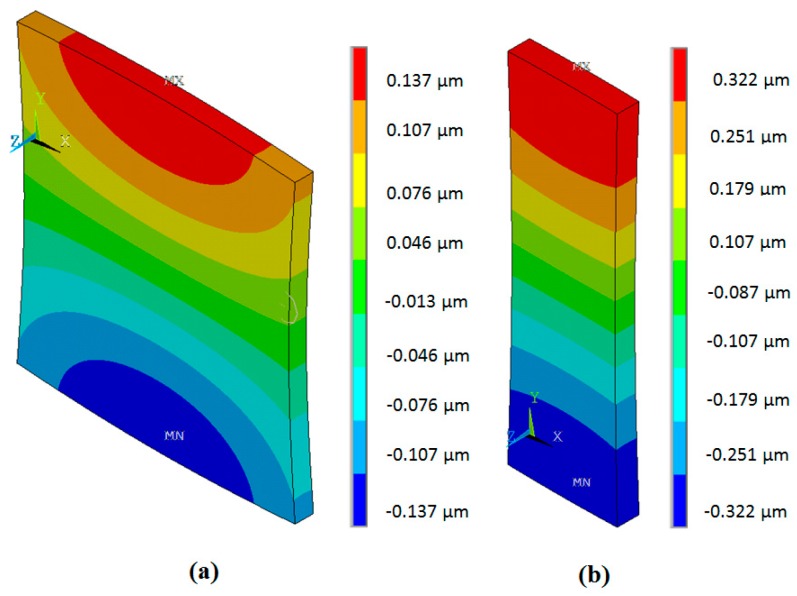
Spatial distributions of the mechanical *y*(2) displacements modulus on the surface of PMN-32%PT elements: (**a**)—15 × 15 × 1 mm^3^, *f*’_r2_ = 37.5 kHz; (**b**)—15 × 5 × 1 mm^3^, *f*”_r2_ = 40.7 kHz.

The simulated spatial distributions of the mechanical *y*(2) displacements modulus on the surfaces of PMN-32%PT elements with different dimensions 15 × 15 × 1 mm^3^ and 15 × 5 × 1 mm^3^ are shown in Figure 7. The displacements are colour coded, e.g., the warmer colours correspond to bigger displacements. The numerical values are given in the corresponding vertical scales shown on the right side of each picture. As obtained displacements are very small in comparison to the dimensions of the crystals, in order to show any changes of the geometry of the crystals, the displacements in Figure 7 are not shown on scale but rather they are increased 6 × 10^4^ times.

From the simulation results, it follows that in the case of strip-like elements, the spatial distributions on the main active (narrow) edge are uniform and the absolute displacement in this case is 2.35 times higher than of the square element. This justifies the selection of PMN-32%PT piezoelectric strips with dimensions 15 × 5 × 1 mm^3^ as active elements for air-coupled ultrasonic transducers.

Performance of the ultrasonic transducers may be significantly enhanced by matching the elements attached to the piezoelectric strips (Figure 4b,c). In order to select the most suitable material various materials such as polystyrene foams AIREX T90.210, AIREX R90.300 (AIREX AG, Sins, Switzerland) and Finnfoam (Finnfoam OY) were investigated. The velocities of ultrasonic longitudinal waves in such materials was not known therefore we have measured them by a through-transmission pulse method at the frequency of 50 kHz in specially prepared samples, the dimensions of which were bigger than the wavelength.

We have found that the matching strips made of Finnfoam material do not help to improve frequency response of the transducer [28,29] therefore, they were not analysed in this paper. The measured acoustic properties of AIREX polystyrene foams are presented in Table 1.

The chosen length of the matching strips was a quarter of the wavelength λ of the ultrasonic wave in the polymeric matching element at the operation frequency. As it is known the velocities of ultrasonic longitudinal waves in bulk materials and strips (and bars) are different, due to the different boundary conditions. In strips, due to the free boundary conditions, the ultrasound velocity is lower than in bulk materials, therefore in order to select the proper length of the matching strips the ultrasound velocity was measured by the through transmission method in strips 1 mm thickness. The results of those measurements are presented in Table 2.

**Figure 8:**
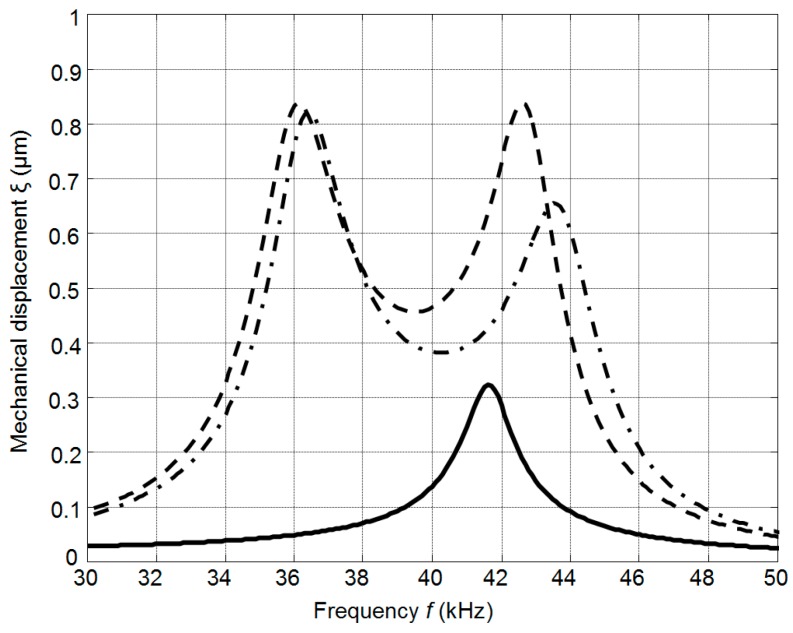
Simulated frequency responses of the single PMN-32%PT 15 × 5 × 1 mm^3^ element mechanical displacements: without matching strip—solid line; with the matching strip (*w* = 1 mm, *R*_g_ = 50 Ω): AIREX T90.210—dashed line; AIREX R90.300—dot-dashed line.

The calculated frequency responses of the mechanical displacements of the active surface of the ultrasonic transducer without matching strip and with the matching strip (AIREX T90.210 and R90.300) are presented in Figure 8. The frequency responses were obtained by calculating the displacements of the central point of the radiating surface by FEM at different frequencies. In all cases the output electric impedance of the electric generator was *R*_g_ = 50 Ω. In the frequency responses presented (Figure 8) two resonance peaks at the frequencies of 36 kHz and 43 kHz exist. They occur due to a coupling phenomenon of two resonant elements—the piezoelectric crystal and the matching strip. The better performance is obtained with the matching strip made of AIREX T90.210 material possessing lower acoustic impedance. From the results presented it follows that there is an improvement of the bandwidth at the −6 dB level and efficiency due to matching of the acoustic impedances of the transducer and air. 

**Figure 9:**
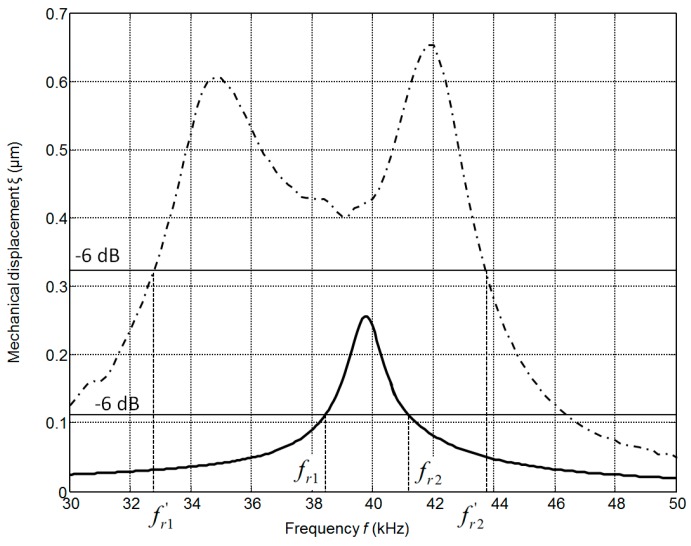
The frequency response of the PMN-PT 15 × 5 × 1 mm^3^ single crystal element without matching strip (solid line) and with the AIREX T90.210 matching strip (dashed line) measured by the laser interferometer.

The frequency responses of the displacements normal to the active surface measured by the laser interferometer OFV-5000 (Polytec) are shown in Figure 9.

**Figure 10:**
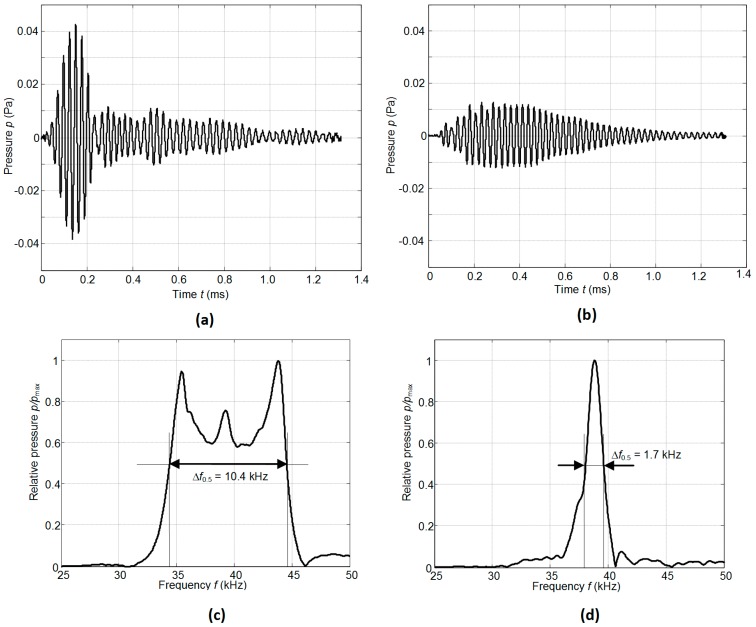
Acoustic pressure pulses in air at the distance 1 mm from the transducer measured by the Brüel & Kjær wideband 1/8” microphone: (**a**)—PMN–32%PT single crystal transducer, *f* = 36.7 kHz; (**b**)—Murata MA40B7 transducer, *f* = 37.9 kHz; (**c**)—spectrum of the signal radiated by the PMN-32%PT transducer; (**d**)—spectrum of the signal radiated by the Murata MA40B7 transducer.

There is some difference (less than 1–2 kHz) between the simulated and measured resonance frequencies because in the simulations we have used data provided by the manufacturer. On the other hand, we have found that there are some variations of the parameters of different crystal plates. The frequency bandwidth Δ*f* = (*f_r_*_2_ − *f_r_*_1_) was estimated at the −6 dB level (denoted by the horizontal lines in Figure 9) from the maximal value of the corresponding transducer. The relative bandwidth Δ*f*/*f_r_* of the single transducer without the matching strip is 0.07 and with the matching strip 0.28, e.g., four times wider. The experimentally determined pulse response of the transducer with the matching strip made of AIREX T90.210 is presented in Figure 10a. For comparison, the pulse radiated by the commercially available MA40B7 transducer (Murata Manucafturing Company, Ltd., Kyoto, Japan) is shown in Figure 10b. The spectra of both recorded signals are shown in Figure 10c,d, respectively. Both transducers were excited by sine bursts of five periods by a function/arbitrary waveform generator (HP 33120A, Hewlett-Packard, Palo Alto, CA, USA) with the central frequencies adjusted to the operation frequencies of the transducers, e.g., close to *f* = 40 kHz. The peak-to-peak excitation voltage in both cases was the same *U*_pp_ = 18 V. The measurements were performed in air using 1/8 inch pressure-field microphone (B&K 4138-A-015, Brüel & Kjær, Naerum, Denmark) which possesses a flat pressure-field frequency response in the frequency range from 6.5 Hz to 140 kHz. From the results presented in Figure 10c,d it is possible to see that the bandwidth of the proposed PMN-32%PT transducer at the −6 dB level is 6.1 times wider than of the Murata MA40B7 transducer.

**Figure 11:**
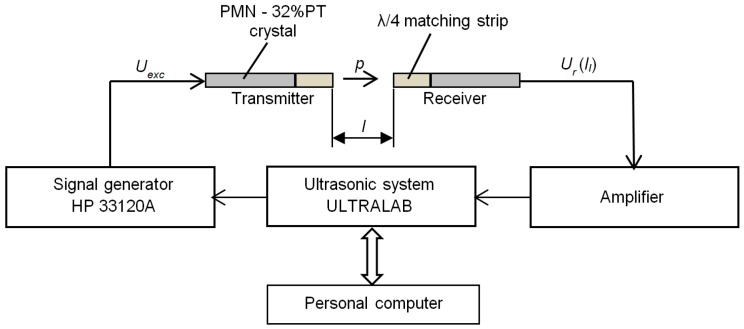
Measurement of transduction losses of air-coupled ultrasonic transducers.

The transduction losses of the developed single crystal transducer with the λ/4 matching strip were estimated using two identic transducers. The measurement principle is shown in Figure 11.

The transmitter was excited by a sine burst with the frequency *f*_r2_ = 41.5 kHz generated by the HP 33120A function/arbitrary waveform generator. The received signal was amplified and stored for analysis by the ultrasonic measurement system (ULTRALAB, Ultrasound Institute, Kaunas University of Technology, Kaunas, Lithuania). The total signal losses *T*(*l_i_*) depend on the distance between transducers *l_i_* and are found from the measured values of the received signal *U*_r_(*l_i_*) and the excitation voltage *U*_exc_:

(1) $$T\left(l_{i}\right)=20\log\frac{U_{r}\left(l_{i}\right)}{U_{\mathrm{exc}}}.$$

The total signal losses consist of the transduction losses *T*_trans_, losses due to diffraction *T*_dif_(*l_i_*) and attenuation *T*_att_(*l_i_*) of ultrasonic waves in air:

*T*(*l_i_*) = *T*_trans_ + *T*_dif_(*l_i_*) + *T*_att_(*l_i_*). (2)

Attenuation in air at 40 kHz is quite low, α = 0.01 dB/cm, therefore it can be neglected [30]. It should be noted that transduction losses *T*_trans_ differently from the diffraction losses *T*_dif_(*l_i_*) do not depend on the distance *l_i_* and it might be exploited for elimination of the diffraction losses.

For this purpose measurement of the normalized diffraction losses $T_{\mathrm{dif}}^{n}\left(l_{i}\right)$ per distance unit, for example, 1 mm, were performed at the two fixed distances *l_i_* and *l*_-*i-*1_ and calculated in the following way:

(3) $$T_{\mathrm{dif}}^{n}\left(l_{i}\right)=\frac{1}{l_{i}-l_{i-1}}\left[20\log\frac{U_{r}\left(l_{i}\right)}{U_{r}\left(l_{i-1}\right)}\right].$$

The total diffraction losses at the distance *l_i_* are found as a sum of the normalized diffraction losses per 1 mm along the whole distance between the transducers *l_i_*:

(4) $$T_{\mathrm{dif}}^{n}\left(l_{i}\right)=\sum_{i=1}^{N}T_{\mathrm{dif}}^{n}\left(l_{i}\right)\left(l_{i}-l_{i-1}\right),$$

where *N* is the number of intervals in which the normalized diffraction losses were measured. Then the transduction losses *T_trans_*_1_ of one transducer are given by:

(5) $$T_{trans1}=\left[T\left(l_{i}\right)-T_{\mathrm{dif}}\left(l_{i}\right)\right]/2.$$

The measured transduction losses *T_trans_*_1_ for one single element transducer are −11.4 dB, what is better than of the most commercially available air-coupled ultrasonic transducers. The transduction losses of the widely used Murata MA40B7 transducer are almost the same, e.g., −11.2 dB; however, the aperture of this transducer is six times bigger than the aperture of our PMN-32%PT single element transducer. In a pulse mode, efficiency of such transducer is four times better than of the Murata MA40B7 transducer (Murata Manufacturing Company, Ltd., Kyoto, Japan) due to the wider bandwidth (Figure 10).

## 5. Investigation of Air-Coupled Ultrasonic Array

**Figure 12:**
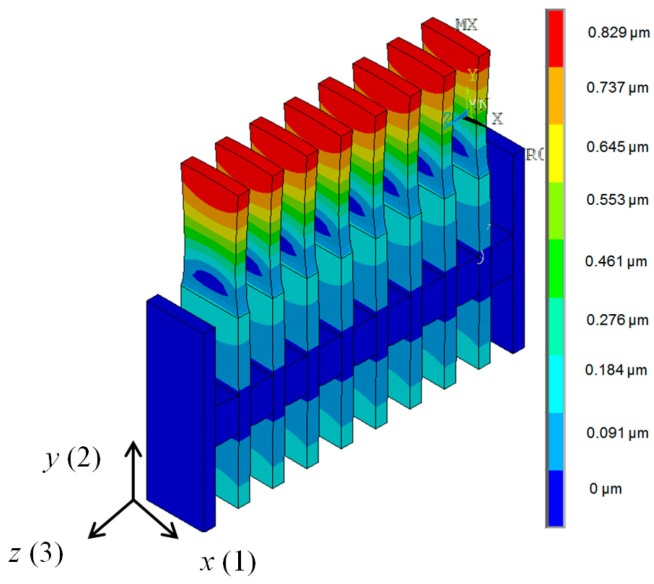
Spatial distributions of displacements of the air-coupled ultrasonic array simulated by FEM at the frequency *f*_r2_ = 42.8 kHz; *R*_g_ = 5 Ω. Amplitude scale is shown on the right in meters.

An ultrasonic array consisting of eight PMN-32%PT crystal strips 15 × 5 × 1 mm^3^ with the λ/4 matching strips operating in the transverse extension mode in the *y*(2) axis direction was developed (Figure 5b). The spatial distributions of the displacements modulus in the *y*(2) direction in the array elements at the frequency *f*_r2_ = 42.8 kHz corresponding to the peak in the frequency response of the array are presented in Figure 12. 

The biggest displacements are on the active surfaces of the matching strips, which are used for radiation of the ultrasonic waves. The displacements of the opposite end of the piezoelectric strips are almost nine times smaller.

**Figure 13:**
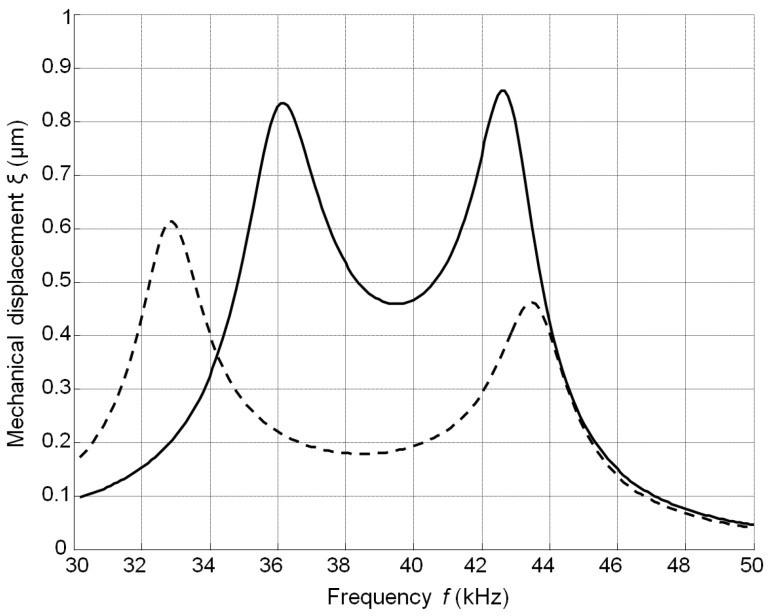
Simulated frequency responses of mechanical displacements of the air-coupled ultrasonic array with the matching strips λ/4 (AIREX T90.210) of different thickness: *w* = 1 mm—solid line and *w* = 3 mm—dashed line (*R*_g_ = 5 Ω).

The frequency responses of the array were obtained in the same way as in the case of a single element. The displacements were calculated for the central element of the array. The frequency responses of the array depend on the thickness of the matching strips. In the case of the thick matching strip (*w* = 3 mm, Figure 13) the frequency response becomes more non-uniform in comparison to the matching strip thickness of which *w* = 1 mm is the same as the thickness of the piezoelectric element. From the simulations follows that the best performance of the air-coupled ultrasonic transducer in terms of bandwidth and transduction losses is obtained with the λ/4 thin (*w* = 1 mm) matching strip made of AIREX T90.210 plastic (AIREX AG, Sins, Switzerland).

**Figure 14:**
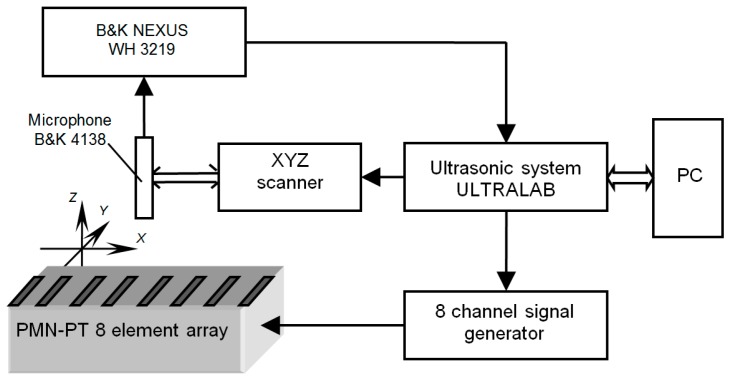
Experimental set-up for investigation of ultrasonic fields radiated by the air-coupled ultrasonic array: PC—personal computer.

Frequency responses of the developed array were measured by the experimental set-up shown in Figure 14. The ULTRALAB ultrasonic measurement system and the multichannel signal generator (Ultrasound Institute of Kaunas University of Technology, Kaunas, Lithuania) formed the electric signal used for excitation of the array elements.

The excitation instant for each element may be controlled individually. The radiated ultrasonic signal was picked up the B&K 4138 type microphone and amplified by the B&K NEXUS WH 3219 amplifier. The three axes X, Y, Z scanner drives the B&K 4138 type microphone in space. A short unipolar electric pulse duration of 0.25 μs excited the eight elements of the developed array. This means that in the 30–50 kHz frequency range it is possible to assume that the spectrum of the excitation signal is uniform. 

**Figure 15:**
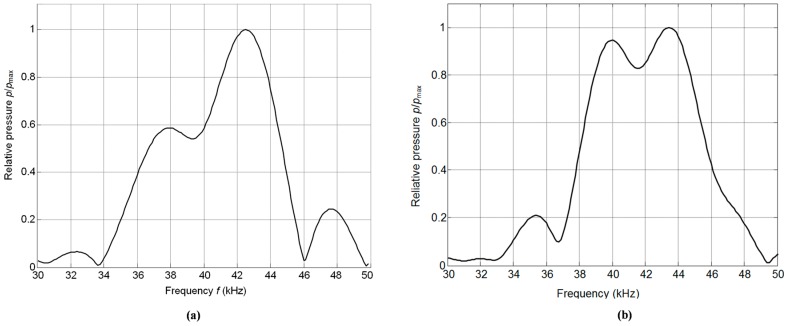
Measured frequency response at the distance 80 mm (**a**) and spectrum at the distance 60 cm (**b**) of the normalized acoustic pressure *p*/*p*_max_ radiated by the array.

The frequency response of the array was evaluated by calculating the spectrum of the received ultrasonic pulse using a fast Fourier transform. The measurements were performed on the symmetry axis of the array in a far field zone, e.g., at the distance 80 mm from the array surface. The results of measurements are presented in Figure 15.

Due to the interference of the ultrasonic fields radiated by individual elements the frequency response is rather smooth and the achieved frequency bandwidth at the 0.5 *p*/*p*_max_ or −6 dB level is Δ*f*_0.5_ = 0.2 *f*_0_, e.g., is quite wide (Figure 15). Here *f*_0_ = 39 kHz is the central frequency of the array.

For applications the frequency spectrum of the ultrasonic signal, which depends also on the electric excitation signal of the array, is more important. In the case of the burst with a rectangular envelope and duration seven periods the measured spectrum of the ultrasonic signal in a far field zone—60 cm from the array is shown in Figure 15b. From the result presented it follows that the obtained spectrum is symmetrical and wide band.

**Figure 16:**
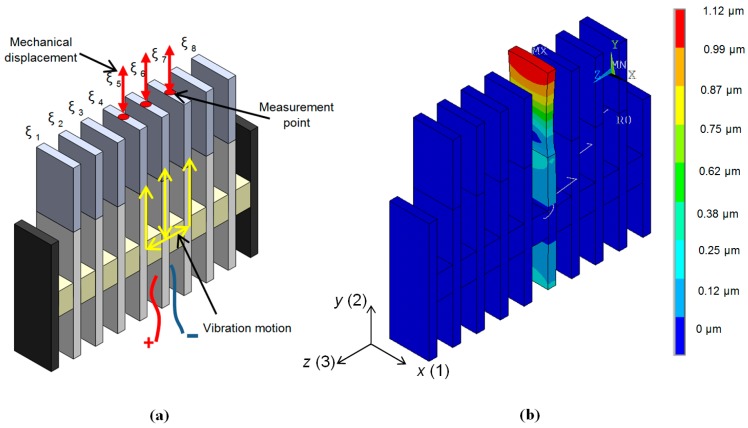
Investigation of the cross-talks levels between neighbouring array elements. (**a**)—explanation of cross-talks between array elements; (**b**)—spatial distributions of displacements of the air-coupled array when excited only element No. 5.

An important parameter characterizing performance of the air-coupled array is the level of cross-talk between neighbouring array elements. Vibrations propagating from the excited element to the adjacent elements via supporting spacing elements (Figure 16a) mainly create the cross-talk. Propagation paths of the vibrations are shown in Figure 16a by yellow arrows. The spacing elements are bonded to PMN-32%PT crystals in the plane corresponding to their vibration node. Due to that and the fact that Finnfoam material from which the spacing elements are made possesses low acoustic impedance (Table 1) the level of cross-talk was minimized.

Investigation of the cross-talk was performed both theoretically and experimentally. In both cases only one element—number 5 in the array—was excited and the displacement amplitudes of the radiating edges (red arrows in Figure 16a) of the excited element 5 and adjacent elements 4 and 6 were determined in the frequency range from 30 kHz to 50 kHz. The excitation voltage was a long sine pulse with the peak-to-peak amplitude *U*_pp_ = 1 V.

**Figure 17:**
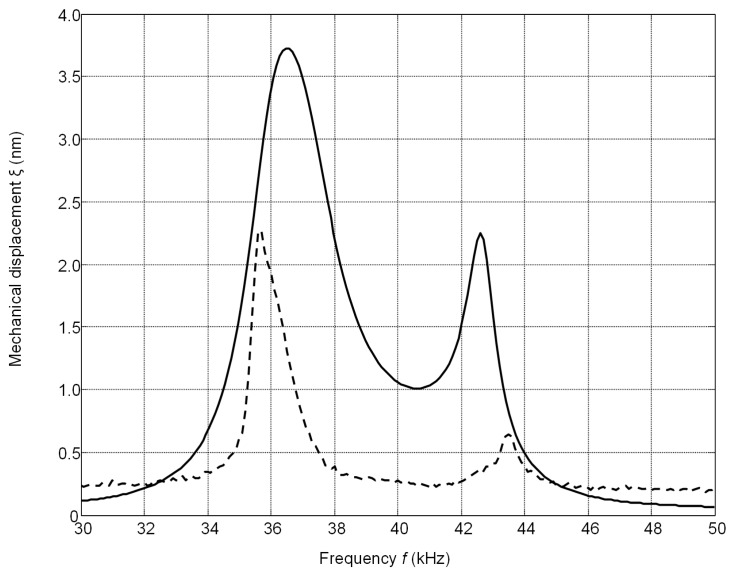
Frequency responses of the mechanical displacements of the adjacent element No. 4 when only element No. 5 is excited: solid line—simulated; dashed line—measured by the laser interferometer.

Theoretical investigation was performed by finite elements modelling. Spatial distributions of displacements in this case are shown in Figure 16b. The simulated frequency response of element No. 4 is represented in Figure 17 by a solid line. The mechanical displacements of this element at the resonance frequency 36.5 kHz were compared with the displacements of element No. 5. The calculated displacements of the elements No. 4 and No. 6 are about 950 times smaller than of the excited the element No. 5. Theoretical results were compared with the results of measurements performed by a laser interferometer OFV-5000 (Polytec). The measured frequency response of element No. 4 is represented in Figure 17 by a dotted line. Comparison with the measured displacements of element No. 5 showed that the measured displacements of element No. 4 are 444 times smaller, what indicates a quite low level of cross-talk.

Another experiment was carried out when all elements of the array are simultaneously excited and the mechanical displacements of the radiating surfaces of the array active elements and the spacing elements were measured at the frequency *f*_0_ = 37.91 kHz. We found that in this case the mechanical displacements of the spacing elements were 20 times smaller than of the excited elements of the air-coupled array.

## 6. Investigation of Acoustic Fields

Application of air-coupled ultrasonic arrays for purposes such as excitation and reception of guided waves or for navigation of mobile robots requires the possibility to control the radiated acoustic fields, e.g., to focus and to scan the surrounding space.

**Figure 18:**
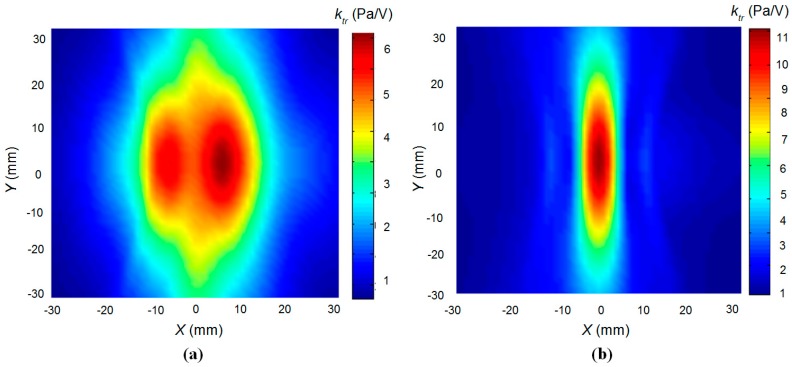
Numerical simulation of the air-coupled array. Unfocused (**a**) and focused (**b**) cases of normalized acoustic pressure at the distance *d* = 20 mm.

The possibility to focus the acoustic beam was checked by a numerical simulation and experimentally. For simulation of the acoustic pressure generated by the array of rectangular pistons, the impulse response method was applied [31,32]. The experiments were carried out by the ULTRALAB ultrasonic measurement system (Ultrasound Institute of Kaunas University of Technology, Kaunas, Lithuania) described above using the 1/8 inch pressure-field microphone B&K 4138-A-015. The simulation results are presented in Figure 18. The cross-sections of the acoustic beam pressure *k*_tr_ = *p*/*U* normalised with respect to the excitation voltage *U* at the distance of 20 mm (intermediate field) of the unfocused and focused array are shown respectively in Figure 18a,b. The maximal normalized acoustic pressure in the unfocused beam is *k*_tr_ = 6 Pa/V and *k*_tr_ = 11 Pa/V in the focused beam, e.g., 1.8 times higher than in the unfocused beam.

**Figure 19:**
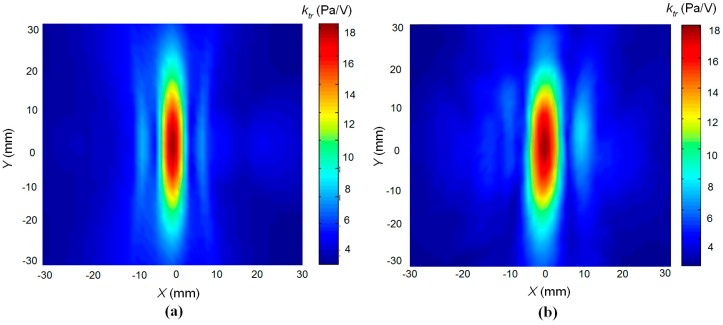
Comparison of numerical simulation and measurements results: (**a**)—simulated acoustic pressure of the air-coupled array at the focus distance 10 mm; (**b**)—measured acoustic pressure of the air-coupled array at the same conditions.

The numerical simulation and measurement results presented in Figure 19 show a good correspondence. It should be noted that the spatial scale in Figure 19 is extended in comparison to the scale in Figure 18. The developed linear array performs focusing only in one *x*0*z* plane; therefore, the focal spot is elongated along the *y* direction. The width of the focal spot along the *x* direction at a distance 10 mm from the array at the −6 dB level is about 4 mm, which corresponds to the wavelength in air, e.g., it is close to a theoretical limit. The measured focal spot looks slightly wider (6 mm) than the calculated one due to spatial integration of the measured acoustic pressure by the finite dimension 1/8 inch microphone.

**Figure 20:**
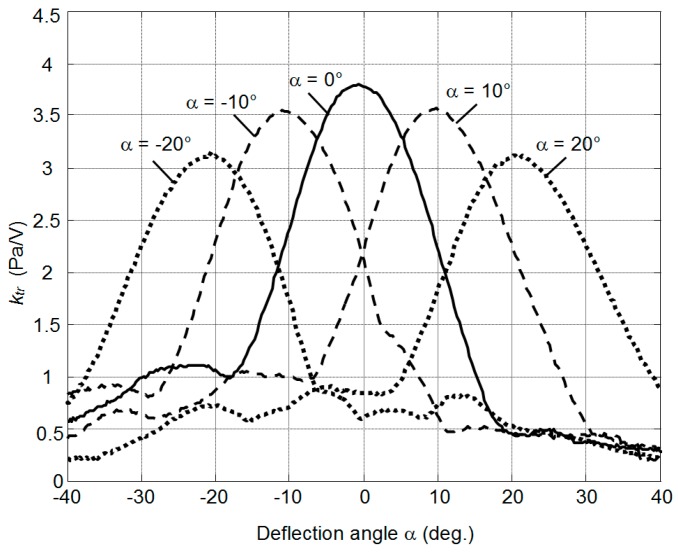
Measured normalized acoustic pressure *k*_tr_ distributions across the ultrasonic beam at the distance 80 mm from the array at different beam deflections angles α.

The possibility to control electronically the deflection of the ultrasonic beam was also investigated. The elements of the array were excited introducing delays of the excitation instants according to a linear law:

τ*_i_* = *t*_0_ ± *i*Δ*t_i_*, (6)

where *t*_0_ is the initial delay, Δ*t_i_* is the delay between adjacent elements and *i* is the number of the array element. The results illustrating the beam steering are shown in Figure 20. In this figure the acoustic pressure *p* normalized with respect to the excitation voltage *k*_tr_ = *p*/*U* is presented.

The results of ultrasonic beam deflection measurement show very good beam steering possibilities in the ±20° range (Figure 20).

## 7. Experimental Investigations

**Figure 21:**
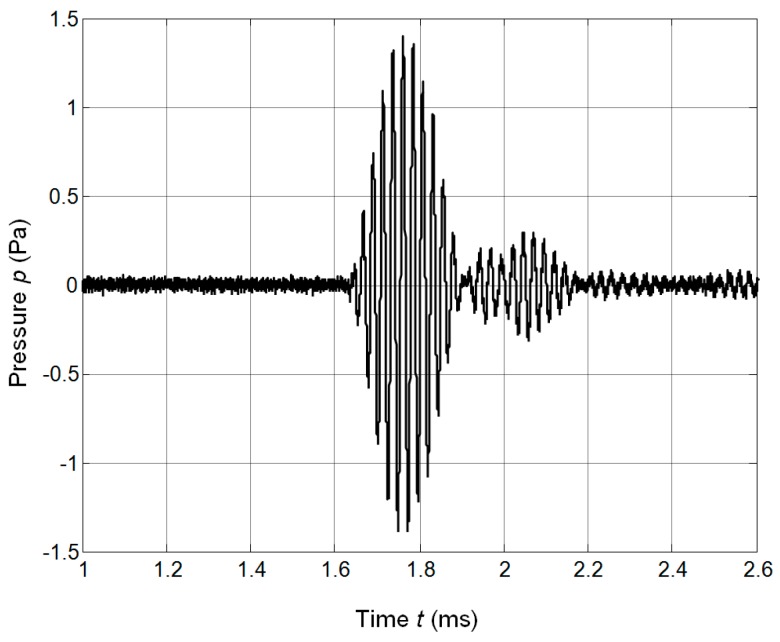
Waveform of the ultrasonic signal radiated by the array and picked-up at the distance 60 cm by the B&K 4138 type microphone.

**Figure 22:**
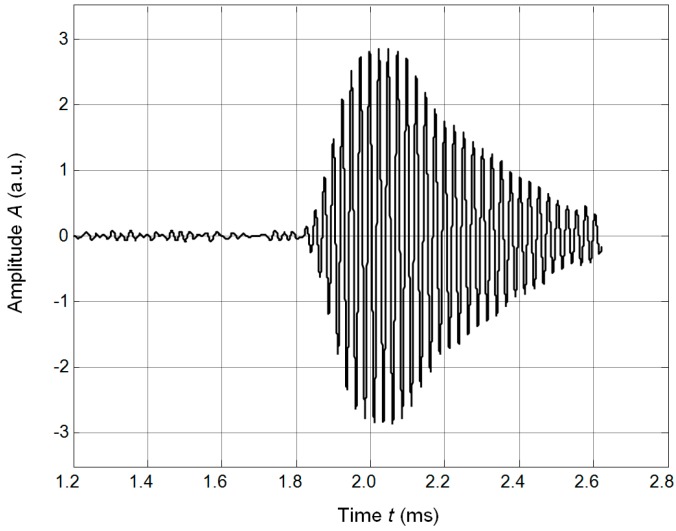
Waveform of the ultrasonic signal reflected from the plane solid reflector at the distance 30 cm.

The performance of the developed array was investigated experimentally. First of all its ability to detect and to perform distance measurements from objects located at some distance was checked. For this purpose, ultrasonic pulses with frequency of 43.1 kHz radiated by the ultrasonic array were recorded at a distance of 60 cm by the B&K 4138 type microphone (Brüel & Kjær, Naerum, Denmark) and amplified by the B&K NEXUS WH 3219 (Brüel & Kjær, Naerum, Denmark) and ULTRALAB amplifiers (Ultrasound Institute of Kaunas University of Technology, Kaunas, Lithuania). The result is presented in Figure 21. After that, ultrasonic pulses reflected from the plane wooden reflector placed at the distance 30 cm from the array were recorded. This distance corresponds to a total propagation path of 60 cm. The results are shown in Figure 22.

**Figure 23:**
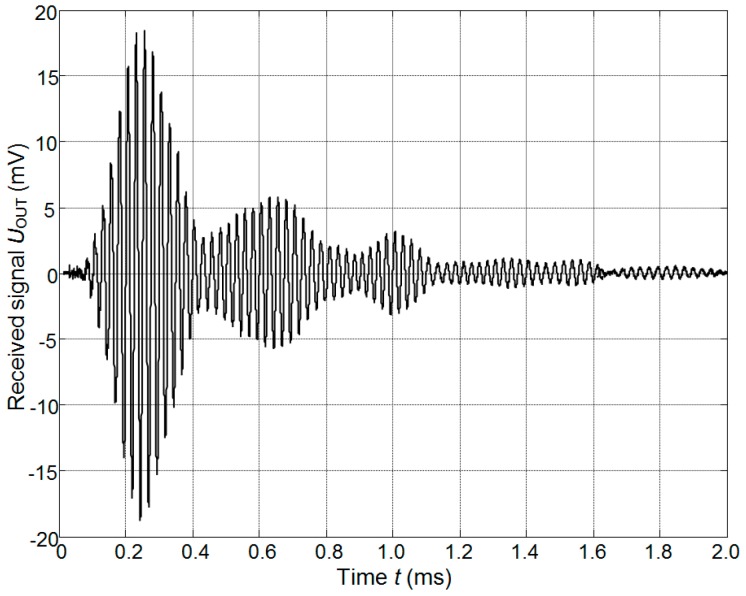
Waveform of the ultrasonic signal in the through transmission-reception mode of the pair of the single element transducers with matching strips.

The performance of the pair of the single element transducers with matching strips facing each other was investigated in a through transmission-reception mode. The transmitter was excited by the rectangular burst of seven periods and a frequency of 41.4 kHz. The ultrasonic signal received by another identical transducer was amplified by the ULTRALB system and is presented in Figure 23. 

**Figure 24:**
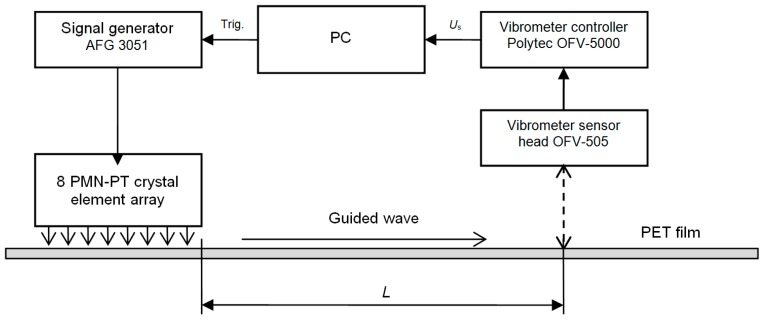
Experimental set-up for investigation of the excitation of the guided wave.

The possibility to excite guided waves in a viscoelastic thin plate was checked using the experimental set-up shown in Figure 24. For experiments, a thin plate of 0.39 mm thickness made of polyethylene terephthalate (PET) was selected. This material is a widely used thermoplastic polymer resin of the polyester family. The air-coupled 8-element array was excited by a 39.4 kHz and 15 periods electric burst. 

**Figure 25:**
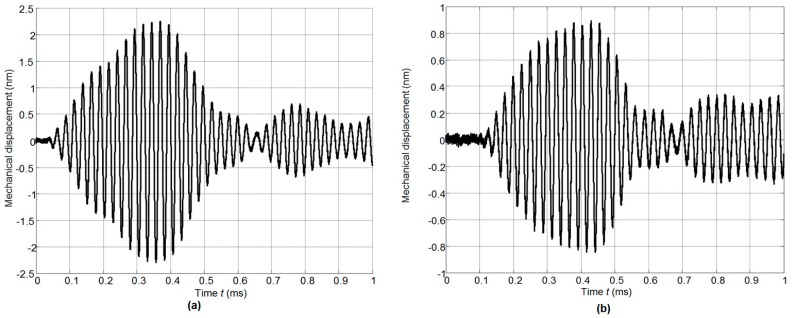
Waveforms of A_0_ mode normal displacements in PET plate recorded by the laser interferometer OFV-5000 at two different distances from the array: (**a**)—*L* = 10 mm; (**b**)—*L* = 40 mm.

The ultrasonic wave transmitted via the air gap between the array and the plate excited the A_0_ mode antisymmetric Lamb wave in the plate. The normal displacements of this mode were recorded by the OFV-5000 (Polytec) laser interferometer and are shown in Figure 25a,b.

The delay of the guided ultrasonic wave was measured while performing measurements at two different distances *L* from the array and the group propagation velocity estimated. The obtained group velocity value was *v* = 405 m/s, which corresponds to a slow A_0_ Lamb wave. The corresponding phase velocity was *v* = 202 m/s. The presented example shows the possibility to excite with the proposed air-coupled array guided ultrasonic waves in plates in the case where the phase velocity of those waves is slower than the velocity in air.

## 8. Conclusions

The performance of air-coupled transducers may be significantly enhanced using as active elements PMN-32%PT single crystals vibrating in the transverse extension mode. Application of this mode enables one to achieve operation frequencies lower than 100 kHz. For radiation, the edge of the crystal plate is exploited. For further improvement of a performance, strip-like thin matching elements made of low acoustic impedance materials such as polystyrene foams may be used. The matching elements improve not only efficiency, but the bandwidth of the transducer and the radiated pulse waveforms as well.

The aperture used for radiation of ultrasonic waves in this case is rather small, therefore an ultrasonic array assembled from individual strip-like elements was proposed. The frequency response and the bandwidth in this case are quite similar to the frequency response of the single elements. Finite element modelling shows that the biggest displacements are obtained on the radiating surfaces of the matching strips, whereas displacements of the opposite end of the piezoelectric element are almost nine times smaller. Theoretical and experimental investigations of the acoustic fields radiated by the eight element ultrasonic array demonstrated not only a good performance of the array in a pulse mode, but also very good possibilities to electronically focus and steer the ultrasonic beam in space.

## Figures and Tables

**Table 1:** Properties of polymers used in matching elements.

Properties	AIREX T90.210	AIREX R90.300
Density *ρ* (kg/m^3^)	200	300
Ultrasound velocity *c* (m/s)	1340	1513
Acoustic impedance *Z* (MRayl)	0.268	0.454

**Table 2:** Acoustic properties of strip-like matching elements.

Properties	AIREX T90.210	AIREX R90.300
Ultrasound velocity *c* (m/s)	1272	1385
Acoustic impedance *Z_s_* (MRayl)	0.254	0.415
λ/4 (mm)	7.6	8.3
